# Supplementary material for: Behaviours involved in the role of victim and aggressor in bullying: Relationship with physical fitness in adolescents
Source: PLoS One. 2021 Nov 4;16(11):e0259087. doi: 10.1371/journal.pone.0259087 (PMC8568104; doi:10.1371/journal.pone.0259087)
Supplement: Attachment — Submitted filename: Comments.docx [file pone.0259087.s002.docx]

Dear Authors,

Priorities I congratulate the authors for their ideas and efforts. The time they spend working is very valuable. Two main objectives are proposed: know the relationships between physical fitness and victimisation and aggression in bullying and know which behaviours associated with aggression and victimisation in bullying are more specifically related to cardio-vascular endurance, muscular strength and flexibility as components of physical fitness.

As major revision I make three considerations

The methodology applied to respond to the objectives is correct, however, from my point of view, additional statistical analyzes are required for a better response. Why do the authors perform a Spearman correlation? In abstract section authors indicate Pearson Correlation. Furthermore, I propose to the authors to carry out two additional tests: Multiple stepwise regression analysis, with the aim of identifying the most related component of physical condition with dependent variables.

One-way ANOVA to analyze whether there are differences in physical condition between subjects with differential points in victimisation and aggression in bullying.

Second, while the study appears to be sound, however, the authors should rewrite their Introduction and Discussion. Introduction: Since previous studies have analyzed the object of study of this manuscript, I consider it necessary to include quantitative results of these studies in the introduction section.

Third: what is the difference with Garcia-Hermoso A, Oriol-Granado X, Correa-Bautista JE, Ramírez-Vélez R. Association between bullying victimization and physical fitness among children and adolescents. Int J Clin Health Psychol. 2019 May;19(2):134-140. doi: 10.1016/j.ijchp.2019.02.006. Epub 2019 Apr 17. PMID: 31193131; PMCID: PMC6517651.

The authors should revise the language to improve readability.

As minor revisions

First paragraph, no relationship between first and second sentence is shown.

Line 24: delete "n"

Line 31: Pearson or Spearmar?

Line 36-40: Conclusions: review subsequent comment regarding conclusions

Line 47: delete “,”

Line 80-83: Can authors justify this argument with a reference?

Line 93: delete " [".

Line 100: “…these focusing on the role of the victim and concluding that good physical fitness may…” ¿Good or high physical fitness? At this point, it would be interesting for readers that authors reported more information.

Line 105-107: Idem, provide more specific contents

Line 118-124: The authors say that: “…descriptive study involved 1035 schoolchildren aged 12 to 19 years…” However, later it is indicated that they are second year of baccalaureate and that those over 17 years were excluded. Clarify

Materials and methods section.

I propose to the authors a modification of the distribution of this section for a better understanding. Include a section on design or experimental design. Below is the description of the participants and later the procedure and statistical analysis.

Line 150: Why did the authors carry out the analysis of body composition with Tanita? What was the protocol used for a correct measurement using es? Where are the results provided?

Line 153: m2

Line 155: substitute “training” for “familiarization”.

Line 156: Three?

Line 188: The authors use Course Navette and Multistage Shuttle Run Test interchangeably, taking into account the original reference to use Multistage Shuttle Run Test. In addition, this test estimate the cardiorespiratory fitness or aerobic fitness not cardiovascular endurance

Line 210-211 I agree with the authors: Given the cross-sectional nature of this study, this prediction can only be interpreted on a theoretical basis and no causal relationship can be established. Therefore, the conclusions have to be reformulated.

Results section.

Table 2: I propose to align the content to the left. Replace “,” for “.” to indicate decimals. Indicate the units in all variables

Table 3: Idem

Line 239: ¿ weight or BMI?

Table 4: Indicate the units in all variables. Confidence intervals can be given in Table 4

Line 248-250: Can the authors confirm that this sentence is correct?

Line 252: “… being higher in boys…” How much?

Line 252: Is correct to age?

In Tables 6 and 7 the authors report the ordinary regression for some questions. Did the rest of the questions show no relationship? This is important for the correct writing of the conclusions and results.

Line 257-258: In this paragraph, results of table 6 are presented. The results are not related to the table results. Authors can rewrite this paragraph for better understanding. Also, it is not necessary to duplicate the results (text and table)

Discussion section.

The discussion section has to be rewritten. The authors abuse a chaining of paragraphs of results without arguing the same. The structure “In relation to...” is reiterative. It is necessary to discuss providing data from previous studies and comparing with those of this research, not just describing.

Conclusion

Do the results support the conclusions? The authors have to rewrite the conclusions, contributing only those that correspond to the results obtained. Furthermore, a correlation is not a cause-effect relationship, so it must be rewritten.

Is the study free of limitations?
